# Impaction of lower third molars and their association with age: radiological perspectives

**DOI:** 10.1186/s12903-018-0519-1

**Published:** 2018-04-04

**Authors:** Soukaina Ryalat, Saif Aldeen AlRyalat, Zaid Kassob, Yazan Hassona, Mohammad H. Al-Shayyab, Faleh Sawair

**Affiliations:** 1 Department of oral surgery, oral medicine, oral pathology, periodontics and oral radiology, faculty of dentistry, The University of Jordan, Amman, Jordan; 2 School of Medicine, The University of Jordan, Amman, Jordan

**Keywords:** Third molar, Mandibular, Impaction, Eruption, Pattern, Extraction

## Abstract

**Background:**

Third molars are the most commonly impacted teeth, and their extraction is the most commonly performed procedure in oral and maxillofacial surgery. The aim of the present study is to describe the pattern of mandibular third molar impaction and to define the most appropriate age for prophylactic extraction of mandibular third molar teeth.

**Methods:**

A total of 1198 orthopantomographs (OPGs) with 1810 impacted lower third molars were reviewed by two authors. The pattern of eruption in relation to patient’s age was examined using standard radiographic points and angles. Statistical analysis was performed using SPSS for Windows release 16.0 (SPSS Inc., Chicago, IL, USA).

**Results:**

In patients older than 20 years, vertical pattern of impaction was the most common (21.4%); while in young patients; horizontal impaction was more common (21.3%). Furthermore, there was a constant pattern of increase in Pell-Gregory ramus class 1 with increasing age, as the prevalence of class 1 was 0% at age 18 years compared to 54.9% at the age of 26 years.

**Conclusion:**

Frequency of vertical impaction of lower third molars was seen more at an older age (> 20 years) in this study, with an increase in the retromolar space. Late extraction of mandibular third molar teeth (i.e. after the age of 20) is therefore recommended when prophylactic extraction is considered.

## Background

Extraction of impacted molars remains one of the most commonly performed procedures in oral and maxillofacial surgery. Third molars are the most commonly impacted teeth, with an average worldwide rate of impaction of 24% [1]. According to a recent review, prophylactic extraction of asymptomatic third molars occurs in disorderly manner without clearly defined criteria [2]. It has been estimated that 54% of mandibular third molars are removed prophylactically without the presence of any subjective symptoms, and 30 - 50% of referred third molars are removed without any valid indications [3, 4]. One reason for this is the difficulty in predicting which impacted tooth will cause complications if left unextracted.

The current general approach in dealing with impacted third molars is on the basis of clinical judgment; periodic evaluation by some clinicians and early extraction by others [2]. Most expected complications following third molar surgery include sensory nerve damage, dry socket, pain, swelling, trismus, infection and hemorrhage [5]. These complications are disturbing for young patients, especially if they become permanent in cases of inferior alveolar or lingual nerve injuries [6]. In addition to factors related to surgery, the position and angulations of third molars are strongly associated with the number and degree of postoperative morbidities [7]. The purpose of the present study of the pattern of eruption of mandibular third molars is to define the most appropriate age for prophylactic extraction of these teeth. Identification of eruption/impaction pattern of mandibular third molar would help to predict whether mandibular third molar will erupt or will remain impacted, therefore might help in clinical decision regarding the best timing of their extraction.

## Methods

The study was approved by the ethical and research committee at the faculty of dentistry, University of Jordan, Approval no. (1558), and has been conducted in full accordance with the world medical declaration of Helsinki.

A total of 4600 orthopantomographs (OPGs) were retrieved, all OPGs taken in the Department of Dentistry at The University of Jordan Hospital between the years 2010 and 2014 were reviewed. A total of 3402 OPGs were excluded for the following reasons; 1) patient age less than 18 years, 2) patient age more than 26 years, 3) patients with craniofacial anomalies or syndromes, 4) poor quality images, 5) presence of orthodontic treatment, 6) fully erupted third molars, 7) third molars with fussed or dilacerated roots, 8) buccaly or lingually angulated third molars, 9) third molars requiring three dimensional assessments. The final sample therefore consisted of 1198 OPGs with 1810 impacted lower third molars (1224 bilateral impactions, 586 unilateral impactions).

**Fig. 1:**
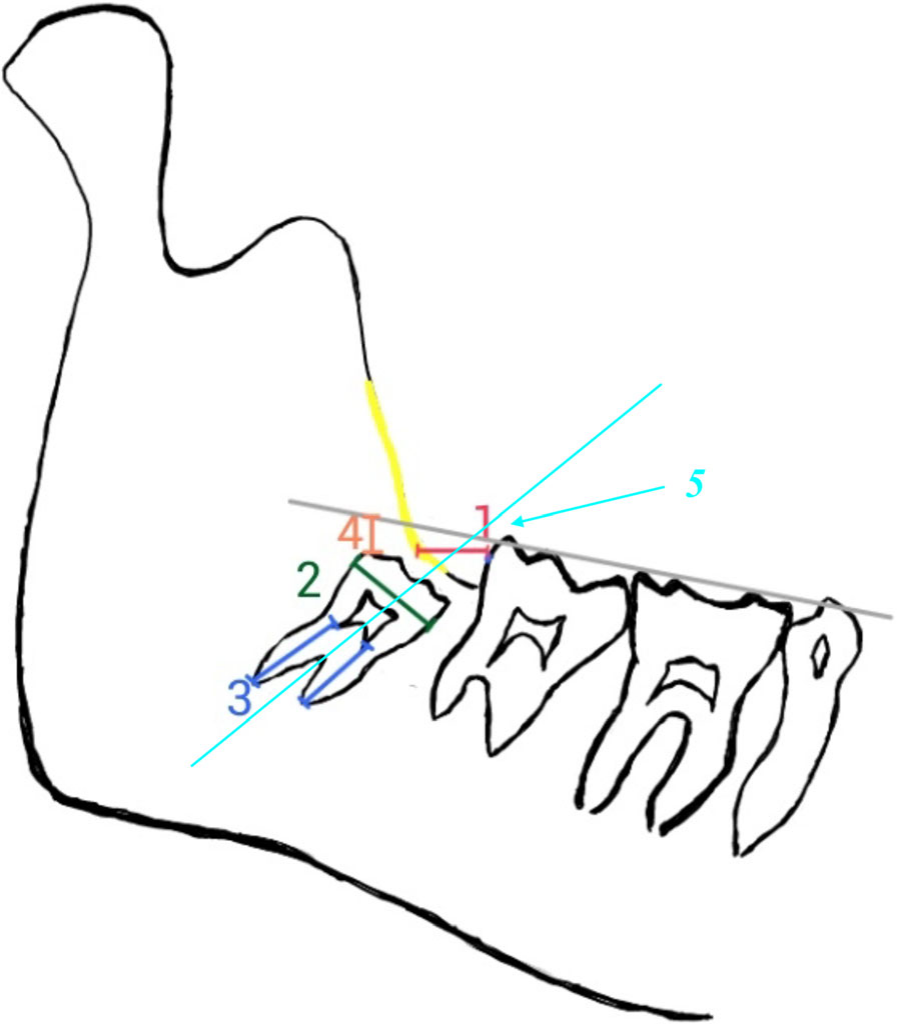
Demonstration of radiographic measurements William Sciller [8]: (1) The retromandibular space, measured between the lines of the anterior border of the ramus to the most distal point of the lower 2nd molar. (2) The length of the horizontal line on the widest area of the crown. (3) The length of the roots. (4) The distance between the highest points of the impacted lower third molar tooth to the occlusal line. (5) Angulation of the third molar refers to the angle formed between dental long axis of the third molar tooth to the occlusal plane

During the period of the study all the patients were handled by the same technician and OPGs were taken by the same machine and processed by the same program (Kodak 8000C, France). Standard panoramic exposures are achieved following standard protocol. Two examiners reviewed all the OPGs and performed the following radiographic measurements according to William Sciller [8], which is presented and simplified in (Fig. 1):

1. The retromolar space. Measured between the lines of the anterior border of the ramus to the most distal point of the lower 2nd molar.
2. The length of the horizontal line on the widest area of the crown.
3. The length of the roots.
4. The distance between the highest points of the impacted lower third molar tooth to the occlusal line.
5. Angulation of the third molar refers to the angle formed between dental long axis and occlusal plane: Horizontal < 20°; Mesioangular = 20–80°, Vertical = 80–100°; Distoangular ≥100°.

**Fig. 2:**
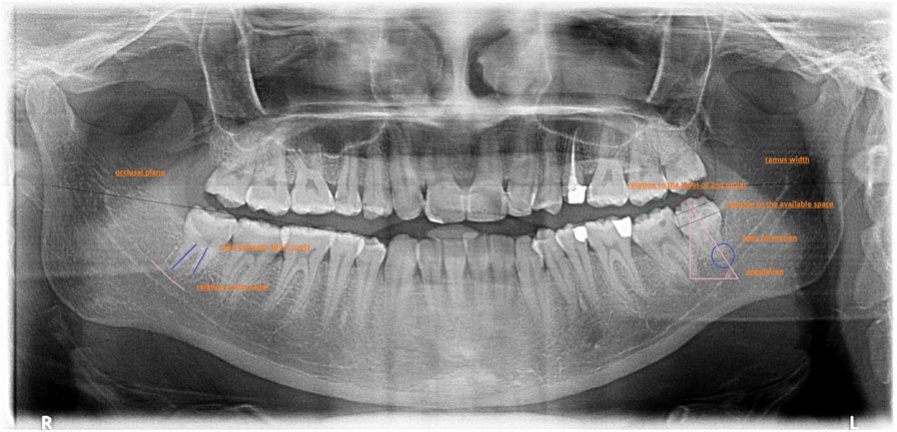
An orthopantograph (OPG) as an example for the measurements of the lower third molar angles

The study used the Kodak software that has built in measuring and marking tools that would calculate the determined angles and lines, and made the OPGs a valuable radiographic technique to be used for location assessment of the lower third molars. (Fig. 2) shows an OPG with the measurements annotated on it.

Statistical analysis was performed using SPSS for Windows release 16.0 (SPSS Inc., Chicago, IL, USA). Descriptive statistics were generated. Student’s t-test and Pearson’s Correlations test (r) were used to examine differences and correlations between groups. Results were considered significant if *P*-values were less than 0.05.

## Results

The sample was composed of 1198 patients (566 males, 632 females) with a total of 1810 impacted lower third molar teeth (1224 bilateral impactions, 586 unilateral impactions) with an age range between 18 and 26 years. The majority of impactions (66.1%) were mesioangular, followed by vertical impactions (18.8%), and horizontal ones (15.1%). The radiographic average width of the impacted molars was 10.1 ± 1.4 mm (range: 8–16.5 mm), (Table 1).

**Table 1:** Features of lower third molar teeth related to age of patients

Age	Impacted lower third molar teeth	Winter’s classification Number (%)			RM space	Mesial root	Distal root	Pell-Gregory ramus Class 1		Pell-Gregory ramus level B	Distance to occlusal line	Distance of apexes to IAC
	Number	H	MA	V	Mean ± SD	Mean ± SD	Mean ± SD	Mean ± SD	(%)	(%)	Mean ± SD	Mean ± SD
18	222	32 (14.4)	165 (74.3)	25 (11.3)	40.0 ± 23.4	6.4 ± 1.2	2.9 ± 1.6	2.1 ± 1.5	0	0	12.5 ± 3.6	1.7 ± 1.1
19	205	42 (20.5)	134 (65.4)	29 (14.1)	37.2 ± 24.8	6.8 ± 1.2	3.4 ± 1.6	2.6 ± 1.4	4.4	2.4	12.6 ± 3.3	2.0 ± 1.2
20	208	61 (29.3)	112 (53.8)	35 (16.8)	37.8 ± 27.0	7.4 ± 1.2	5.0 ± 1.7	3.6 ± 1.4	8.7	8.7	11.7 ± 3.2	2.3 ± 1.3
21	203	20 (9.9)	135 (66.5)	48 (23.6)	49.0 ± 27.7	7.8 ± 1.2	5.5 ± 1.6	4.9 ± 1.7	13.8	25.6	10.4 ± 3.3	2.4 ± 1.2
22	202	35 (17.3)	122 (60.4)	45 (22.3)	43.8 ± 28.1	8.4 ± 1.3	6.0 ± 1.8	5.7 ± 1.7	25.7	31.7	9.9 ± 3.4	2.6 ± 1.3
23	217	22 (10.1)	133 (61.3)	62 (28.6)	49.8 ± 28.0	8.8 ± 1.3	7.0 ± 1.8	6.9 ± 1.7	33.2	52.1	7.6 ± 3.4	2.6 ± 1.2
24	205	20 (9.8)	145 (70.7)	40 (19.5)	45.8 ± 25.5	9.0 ± 1.2	8.1 ± 1.7	7.8 ± 1.5	38.0	48.8	7.7 ± 3.4	2.6 ± 1.2
25	166	18 (10.8)	122 (73.5)	26 (15.7)	43.6 ± 24.4	9.6 ± 1.2	9.9 ± 1.4	9.6 ± 1.4	45.8	47.0	8.2 ± 3.5	2.7 ± 1.2
26	182	23 (12.6)	128 (70.3)	31 (17.0)	44.2 ± 26.3	10.0 ± 1.3	9.9 ± 1.4	9.6 ± 1.5	54.9	54.9	7.2 ± 3.4	2.8 ± 1.2

*RM* retromolar space, *IAC* inferior alveolar canal

The prevalence of vertical impaction in patients older than 20 years old was significantly higher (21.4%) and the prevalence of horizontal impactions was significantly lower (11.7%) than that in younger patients (14.0%) and (21.3%), respectively (*P* < 0.001). The third molar angulation changed significantly between age groups, however, there was no constant pattern from year to year. When age groups were studied, the angulation of lower third molar teeth was significantly higher in patients older than 20 years old (46.2 ± 26.8) compared to younger patients (38.4 ± 25.1), (*P* < 0.001).

A constant pattern of an increase in the retromolar space was noticed with increasing age of patients (*P* < 0.001), the space was close to the average width of the lower third molar teeth after the age of 25 years. This was reflected in a constant pattern of increase in Pell-Gregory ramus class 1 with increasing age, as prevalence of class 1 was 0% at age 18 years compared to 54.9% at the age of 26 years.

**Fig. 3:**
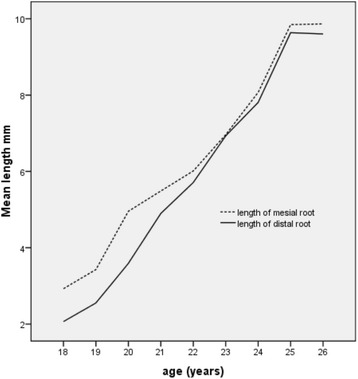
Increase in length of mesial and distal roots of lower third molar teeth with age

The mesial and distal roots’ lengths increased significantly with age as shown in (Fig. 3), (*P* < 0.001). This was revealed by a constant pattern of decrease in the distance of the tooth to the occlusal line with increasing age of patients (*P* < 0.001) and a statistically significant increase in the prevalence of Pell-Gregory ramus level B with increasing age (*P* < 0.001).

## Discussion

The main referral center for third molar teeth extraction for university students, of which the age group range of this study was selected, is the maxillofacial department at our university hospital. The decision of extraction of third molar teeth may be affected by the surgeon’s opinion on the eruption potential of the tooth. A tooth that may appear impacted at the age of 18 years may have as much chance as 30 to 50% of erupting fully, except horizontally impacted molars [9]. The selected group of age (18 years to 26 years) that were included by the study covers the age range that most likely to show significant lower third molar movement [9].

The study showed no significant differences between male and female patients in all calculated values (*p* > 0.05). This finding supported by several studies, except that they found an earlier eruption of third molars compared with males [10]. The lower third molar teeth in this study showed a significant increase in angulations between the ages 20–23 years, in which studies from Europe and United States of America had similar findings [11–13].

The mesioangular impaction was the most frequent impaction by 66.1%, while the horizontal impaction was the least common 15.1%. mesioangular impaction was also found to be the most common in Turkey, Singapore, and China [14–17]. In Sweden, vertical impaction was found to be the most common [18], whereas the vertical impaction was 18.8% in our population.

More vertically angled third molars are seen in older age group, this angulation is the significant point the surgeon looks for when deciding to extract, taking into consideration patient’s age. In this study, the prevalence of vertical impaction in patients older than 20 years was significantly higher (21.4%) and the prevalence of horizontal impactions was significantly lower (11.7%) than that in younger patients whose incidences were (14.0%) and (21.3%), respectively. Additionally, as found by others [19, 20], a constant pattern of an increase in the retromolar space was noticed with increasing age of patients (*P* < 0.001). This was reflected in a constant pattern of increase in Pell-Gregory ramus class 1 with increasing age, which will have a big effect to reduce surgical complications since the tooth may favor eruption. This signifies the importance of re-evaluating patient’s radiograph considering the possible changes of impacted lower third molar teeth that could occur with time, especially for younger patients [1]. Moreover, third molars remain impacted after the age of 25 may still change in position afterward [21].

We believe this study has limitations that should be considered in future projects. Following patients’ clinical and radiological data in a cohort study design or retrospectively in a case-control design will provide a quality evidence on the prophylactic removal, complications, and outcome of third molars.

## Conclusion

Frequency of vertical impaction of lower third molars was seen more at an older age (> 20 years) in this study. Many radiographic readings in patients older than 20 years are in favor of eruption compared with younger patients, including angulation of lower third molar teeth and the increase in retromolar space.
